# Lewis’ law revisited: the role of anisotropy in size–topology correlations

**DOI:** 10.1088/1367-2630/16/1/015024

**Published:** 2014-01-21

**Authors:** Sangwoo Kim, Muyun Cai, Sascha Hilgenfeldt

**Affiliations:** Mechanical Science and Engineering, University of Illinois at Urbana-Champaign, 1206 W Green Street, Urbana, IL 61801, USA

## Abstract

Since F T Lewis’ pioneering work in the 1920s, a linear correlation between the average in-plane area of domains in a two-dimensional (2D) cellular structure and the number of neighbors of the domains has been empirically proposed, with many supporting and dissenting findings in the ensuing decades. Revisiting Lewis’ original experiment, we take a larger set of more detailed data on the cells in the epidermal layer of *Cucumis*, and analyze the data in the light of recent results on size–topology correlations. We find that the correlation between the number-of-neighbor distribution (topology) and the area distribution is altered over that of many other 2D cellular systems (such as foams or disc packings), and that the systematic deviation can be explained by the anisotropic shape of the *Cucumis* cells. We develop a novel theory of size–topology correlation taking into account the characteristic aspect ratio of the cells within the framework of a granocentric model, and show that both Lewis’ and our experimental data is consistent with the theory. In contrast to the granocentric model for isotropic domains, the new theory results in an approximately linear correlation consistent with Lewis’ law. These statistical effects can be understood from the increased number of configurations available to a plane-filling domain system with non-isotropic elements, for the first time providing a firm explanation of why Lewis’ law is valid in some systems and fails in others.

## 1. Introduction

Cellular matter can be loosely defined as a set of individual domains that fill space in typically two dimensions (2D) or three dimensions (3D), either without gaps or with a continuous phase between the domains that takes up only a small fraction of the volume. Even if the continuous phase fraction is high, as it is between packed grains or beads, there are ways to define a space-filling domain structure around the grains by constructing space-filling polygons or polyhedra through Voronoi tessellation [1], Laguerre tessellation [2] or the navigational map [3, 4].

**Figure 1:**
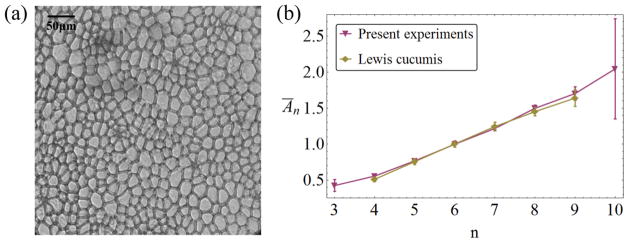
(a) Experimental image of a cross-section of cucumber epidermal tissue. This sample contains about 360 cells of which the neighbor relations can be determined. The image demonstrates both the significant polydispersity of the sample and the elongated shape of most cells. (b) Experimental data for the average area *Ā_n_* of cells with *n* neighbors (Lewis’ law) from the original publications by Lewis [11, 14] (diamonds) and the present results (triangles down). The results from Lewis’ two publications [11, 14] are essentially indistinguishable, so the average of the two results is plotted here. Error bars are 95% confidence intervals.

The domain structure depends on the properties of the individual objects which it is made of, in particular on their size distribution and various properties associated with their shape. Moreover, the degree of *order or disorder* plays a crucial role: regular packings of equal-sized grains give rise to periodic space-filling polygonal structures, but the same grains can also fill the space in a random fashion [5–9]. In this disordered case, information about the structure must be statistical in nature, but is far from random. For a long time, researchers have asked questions about the quantitative description of such statistics, and to what extent they can reflect mechanical, physical or biological properties of the individual domains or cells, and even the history of the formation of the structure as a whole. Of particular interest have been properties called ‘topological’ in the community—those associated with the number of neighbor domains *n* of individual domains. The statistics of *n* shows a number of intriguing correlations with that of the domain size—for 2D systems, the (projected) area *A*. One of the first of these observations concerned epidermal tissue of the fruit of the cucumber (*Cucumis sp*.) by the American biologist Lewis [10, 11]. The epidermis is a single columnar layer of cells directly under the cucumber’s skin, so that a cross-section parallel to that skin shows a polygonal domain pattern of cells in 2D (figure 1). Of the careful measurements of cell size, shape and topology Lewis published in a series of papers in the 1920s and 1930s [11–14], one result in particular intrigued researchers: Lewis had found [11, 14] that the average area of cells with the same number *n* of neighbors, *Ā_n_*, grew approximately linearly with *n* (figure 1(b)).

**Figure 2:**
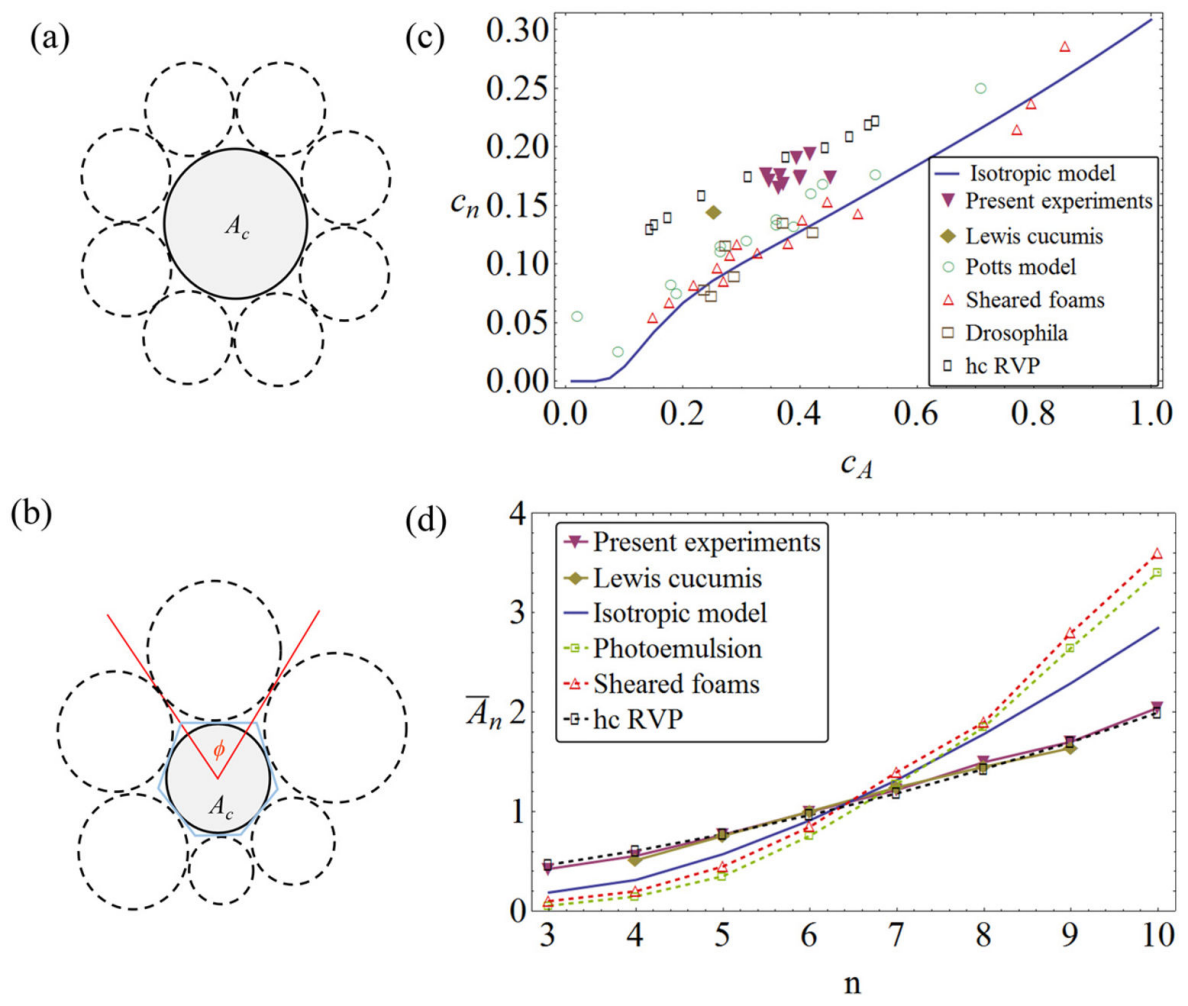
(a) A central disc surrounded by average-sized discs, supporting a nonlinear *Ā_n_*(*n*) law [7, 9]. (b) GM model for polydisperse discs taking up angles *ϕ* around a central disc. (c) Relation between neighbor distribution width *c_n_* and area distribution width *c_A_* for various experimental, simulational and theoretical systems. Potts model simulation data adopted from [20]; sheared foam experiments from [20]; *Drosophila* tissue data from [23], analysis from [7]; and simulations of random Voronoi tilings with hard-core exclusion radii from [24]. Value of *c_A_* for Lewis’ cucumber data [11, 14] estimated, see text. Note that neither the random Voronoi polygon (RVP) data nor the cucumber experiments conform to the results of the isotropic disc theory (solid line). (d) Different systems show significantly different *Ā_n_*(*n*) curves. The linear Lewis’ law is observed for the present cucumber data as well as those of Lewis [11, 14], and also for some RVP simulations (rectangles correspond to the data set of [24] with *c_A_* ≈ 0.49). By contrast, the nonlinear size–topology relation established from the disc model (solid line, [7]) is seen in other experiments, e.g. photo emulsion data from Lewis [14] (squares) and sheared foams [20] (triangles up).

While the qualitative statement of *Lewis’ law* is intuitive (a cell with more neighbors tends to be larger), its linearity is remarkable and even counterintuitive. Figure 2(a) shows a simple argument for guessing a *Ā_n_* law by drawing ‘typical’ (i.e. average-sized) objects as neighbors of a central object of variable size. If we take the average-size objects to have area *Ā* = 1, each takes up a section of length *L̄* = 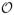
(1) of the central object’s perimeter. As there are *n* neighbors, this central object perimeter must be *L_n_* ~ *nL̄*. But areas scale as the squares of perimeters, so the central object area would be *A_n_* ~ *n*^2^, in contradiction with Lewis’ findings, if *A_n_* from this argument is taken as representative for the average *Ā_n_*. What is wrong about this idea? Are there neighbor correlations that make the idea of ‘average’ neighbors untenable? Is it important to take into account positional disorder [9]?

We revisit Lewis’ experiment to address such questions. In a broader sense, however, Lewis’ law is still a long-standing unsolved empirical finding, which has been reportedly observed not only in diverse systems of cellular matter (living and inanimate) [15–18], but also has been challenged a number of times over the past decades, as it was not able to describe correlations in many other systems (again, living and inanimate) [7, 15, 19, 20]. A fresh look at the issue appears promising because of our group’s recent progress in quantifying a variety of size–topology correlations in 2D and 3D cellular matter [7, 9] using a simple theoretical model.

Section 2 will review this model as it was developed for isotropic objects and successfully applied to a wide range of systems. Section 3 shows our new experimental data and the quantitative characteristics of the cucumber tissue, such as the anisotropy of the cells. In section 4 we derive a new granocentric model (GM) for 2D anisotropic objects. Section 5 shows how the results from the model compare with experiments, and how they contrast with those from previously derived models. Conclusions are presented in section 6.

## 2. The granocentric model

When studying physical, geometrical, and statistical properties in a disordered medium, a paramount question is always whether the structure underlying the properties is the result of long-range spatial correlations (as are e.g. encountered near phase transitions), or whether it can be understood by essentially local properties of the constituting elements and their neighborhoods. We do not attempt to discuss this deep question here, but point out that many statistical features of 2D and 3D complex structures encountered in the experiments have been successfully explained by purely local models recently. In particular, the GM [4, 8, 21] has been developed specifically to explain neighbor statistics in 3D packings and tilings of grains and droplets. In our revisiting of Lewis’ experiment, we are interested in a 2D system and use a 2D version of the GM as a starting point, which was recently developed and tested in our group [7, 9, 22].

In this simplest version, individual objects are replaced by circular discs (figure 2(b)), so that a central disc of area *A*_c_ is surrounded by a statistical ensemble of discs drawn from a size distribution with probability *P*(*A*) and mean size *Ā* = 1. These surrounding discs are supposed to touch the central disc, and take up angles *ϕ* around it. Note that the discs can also be interpreted as templates for polygonal tiles (figure 2(b))—between two discs, an edge can be constructed by a variety of algorithms (for polydisperse discs, the Laguerre or navigational map constructions give unambiguous interfaces between the discs). Given *P*(*A*), a conditional probability *P*(*ϕ*|*A*_c_) can be derived, and from that the probability of the central disc having *n* neighbors, *P*(*n*|*A*_c_). If the central disc is no different than the others, the unconditional probability of having *n* neighbors then simply follows as *P_n_* = ∫ *P*(*n*|*A*_c_) *P*(*A*_c_) d*A*_c_ [7]. If this local computation is to be representative of the statistics of a plane-filling arrangement of objects (discs or polygonal tiles constructed from them), the local computation has to fulfill Euler’s theorem, which—for 2D polygonal tilings—states that the average number of neighbors in the (infinite) ensemble must be *n̄* = 6. In the 2D GM, this condition can be fulfilled either by introducing a stand-off distance between the surfaces of the discs or—mathematically more elegant—by introducing a universal modification to the maximum available angle around each disc (see [7] for details). This theory explains a number of previously empirically known correlations between the *P*(*A*) and *P_n_* distributions, in particular (i) the correlation between the widths of the two distributions, shown in figure 2(c) as a relation of the coefficients of variation *c_A_* and *c_n_*, and (ii) the analog to Lewis’ law, i.e. *Ā_n_* versus *n* (figure 2(d)). The latter is easily obtained from *P*(*n*|*A*_c_) through Bayes’ theorem, and shows a pronouncedly *nonlinear* growth with *n*—roughly in agreement with the *Ā_n_* ∝ *n*^2^ guess of the introduction, though closer inspection shows a somewhat more complicated law [7].

A large variety of experimental and simulational systems conform to the results of the disc model, including the results of various foam experiments, disc packings, *Drosophila* epithelial tissue or Potts model simulations [7, 20]. However, there are exceptions: simulations of RVP tilings do not agree with the theory, and neither do the results of Lewis’ original work, see figures 2(c) and (d). Note that the deviation is of the same kind in all these cases: a larger width of neighbor distribution *c_n_* for a given *c_A_*, and a Lewis’ law correlation closer to a linear law. In [7], it was speculated that, at least for the RVP systems, these deviations can be explained because the domains are not isotropic, i.e. their polygonal outlines are considerably more elongated than those constructed around a circular disc. In the case of RVP tilings, this is due to the lack of any interfacial energy in the individual domains (which are simply mathematical constructs here). By contrast, soap bubbles are dominated by interfacial energy contributions and retain a strong tendency, even in a foam, to remain individually compact and isotropic in order to minimize the individual surface area and thus energy. For the *Cucumis* data of Lewis, it is less obvious whether such an explanation holds, in particular as Lewis did not include comprehensive sketches or image material of his samples. Thus, we decided to acquire such data ourselves.

## 3. Experiments

### 3.1. Materials and methods

English slicing cucumber cultivars of *Cucumis sativus* of length 10–20 cm were obtained. We made no attempt to distinguish between growing and mature fruit (Lewis concentrated on growing cucumbers in [11] and fully grown fruit in [14], but did not specify any particular cultivar in either case). Nevertheless, we obtained consistent results from all the samples, and it is likely that the fruits were not in a state of vigorous growth (see below). Thin sections of the epidermis were prepared parallel to the outer surface of the cucumber, so that the columnar cells appeared as polygons under an inverted microscope (Olympus IX71). Soaking in diluted acetic acid for 48 h removed the green chlorophyll color without changing cell morphology. For enhanced contrast of cell walls, the tissue was stained with Toluidine Blue (Carolina Biological), exposing it to the dye for about 30 min before rinsing. We did not attempt to fix the tissue, which might have distorted or shrunk the cell shapes. Micrographs (figure 1(a)) were taken at 20× or 40× magnification and the resulting images analyzed with CellProfiler [25, 26]. Data was processed from ten samples of nine different cucumbers, with each sample containing between 250 and 800 entire cells in the field of view (i.e. cells whose neighbor number *n* could be determined). The total number of cells analyzed was 4243. The samples were generally taken near the stem end of the cucumber, because there the density of stomatal cells [27] was low. In other sections of the fruit, stomata disrupt the uniform pattern of the epidermis and need to be either analyzed separately or excluded from the sample (Lewis does not mention this difficulty in any of his publications). We did not observe unambiguous examples of cells undergoing divisions, and conclude that dividing cells are a very rare occurrence in our samples (i.e. the fruit is growing slowly or not at all, and the cells can be interpreted as resting or quiescent [14, 28]).

### 3.2. Image analysis

**Figure 3:**
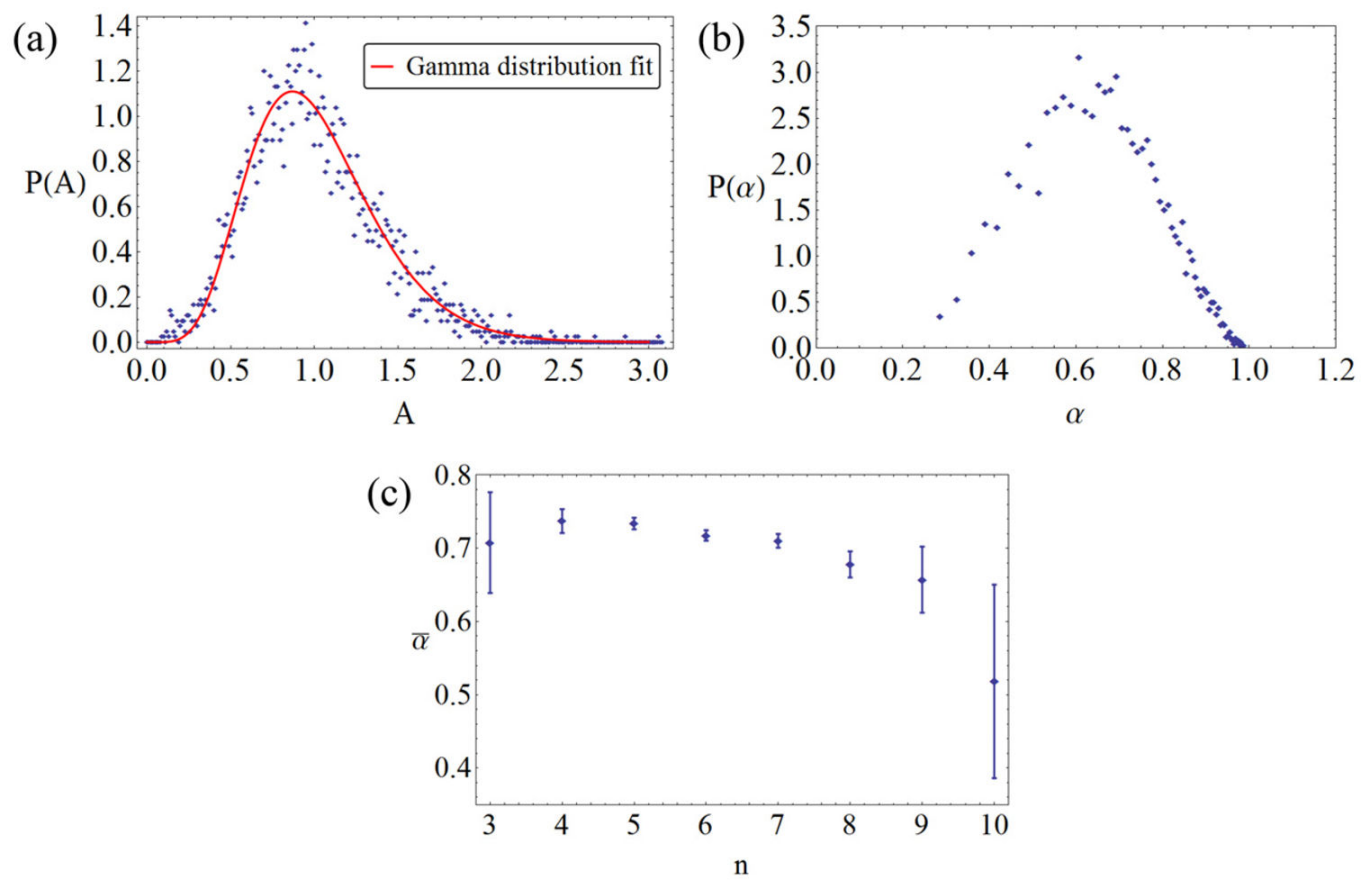
(a) Binned experimental data of area probability distribution (symbols) and gamma distribution fit (solid line), with a coefficient of variation *c_A_* = 0.38. (b) Binned experimental data of probability distribution of cell aspect ratios. (c) Dependence of aspect ratio on number of neighbors; error bars are 95% confidence intervals.

CellProfiler provides data such as the number and identity of neighbors, the cross-sectional area *A*, and the eccentricity *ε* of the cells, the latter being defined as the eccentricity of an ellipse that has the same area and same second area moment as the actual cell [25]. For the purposes of the theory developed later, we translate the eccentricity into an aspect ratio *α*, defining it as the ratio of minor and major axes of this ellipse, so that $\alpha =\sqrt{1-\varepsilon^{2}}\leq 1$. Figures 3(a) and (b) show the probability distributions of the areas *P*(*A*) and the aspect ratios *P*(*α*), respectively. The *P*(*A*) distributions of the individual cucumber samples differ significantly, though not greatly, from each other, but all are well approximated by a gamma distribution

(1) $$P(A)=\frac{1}{\Gamma (c_{A}^{-2})}c_{A}^{-2c_{A}^{-2}}A^{c_{A}^{-2}-1}\exp\;\left(-A/c_{A}^{2}\right),$$

where the mean has been set to *Ā* = 1, and the experimental coefficients of variation *c_A_* for the different samples range between 0.36 and 0.45. The overall distribution in figure 3(a) is described by an average value of *c_A_* ≈ 0.38. The *P*(*α*) distribution (figure 3(b)) is strongly peaked around the mean value *ᾱ* ≈ 0.7. This confirms the visual observation (cf figure 1(a)) that the cucumber cells are pronouncedly anisotropic and elongated. This makes them qualitatively similar to the domains of RVP tilings, though less extreme in shape: translating second-moment data of Poisson RVP domains [1, 29] into aspect ratios leads to a mean of about *ᾱ*_RVP_ ≈ 0.44.

Figure 3(c) demonstrates that the aspect ratio does not strongly correlate with the number of neighbors. While cells with larger *n* tend to be slightly more anisotropic, this effect only appears pronounced for neighbor numbers *n* ≥ 9, where the rareness of these cells (less than 1% of the total) causes large error bars. Assuming a uniform aspect ratio *α* = *ᾱ* for all cells will be our zeroth-order assumption in the model described in the next section. From the neighbor data, the probabilities *P_n_* are determined, again with slight variations from sample to sample, the coefficient of variation *c_n_* ranging from 0.168 to 0.194. The overall average number of neighbors is *n̄* ≈ 5.9995 which is in very good agreement with Euler’s theorem.

When plotting the (*c_A_*, *c_n_*) data points from the experiments in the correlation graph figure 2(c), we notice that they fall between the GM predictions and the RVP data—which is intuitive if the larger *c_n_* values are correlated with larger *α* (anisotropy) of the domains. Note that Lewis’ original publications [11, 14] contain the *P_n_* distribution, but not the full *P*( *A*) distribution, and thus did not include a value for *c_A_*. In order to be able to present this data in the same graph, we estimate Lewis’ *c_A_* as follows: the original paper [11, 14] reports a ‘range’ of areas for cells with a given *n*, together with the average area of these cells. Assuming a normal distribution for the areas of each *n*-neighbor class of cells, we can determine how likely it is that the number of cells in Lewis’ sample (e.g. he took into account the size of *N*_5_ = 100 resting cells with five neighbors in the corresponding range) will *all* fall within the given range, if we assume *c_A_* to have a given value. Increasing *c_A_* from zero, this probability goes from 1 (certainty) to 0 (almost certainly at least one cell will be outside the range). Demanding all neighbor classes of cells to have at least a 50% probability of obeying the range, we obtain $c_{A}^{\text{Lewis}}\approx 0.26$ as the most likely estimate. This is considerably smaller than our samples, but Lewis’ neighbor distribution width is also significantly smaller (*c_n_* ≈ 0.145). These values are the result of averaging data from [11, 14], but the individual values from the two publications differ very little from each other. The resulting data point again lies between disc and RVP predictions in figure 2(c). The discrepancy between Lewis’ *Cucumis* data and ours could be due to a number of potential differences between the samples (unfortunately, no detailed information is available in Lewis’ papers): (i) the fruits could be from different species of *Cucumis*; (ii) if of the same species, they could be different cultivars; (iii) at least some of Lewis’ tissue samples were growing and proliferating, while ours is practically quiescent; and (iv) Lewis may have included stomatal tissue in his samples, which we discarded. Nevertheless, the general finding of a 2D tissue with a relatively larger neighbor distribution width than an equally polydisperse isotropic domain system is common to all samples and results. In order to test our hypothesis, that this effect is due to the anisotropy of the domains, we developed a novel modeling approach.

## 4. A model for anisotropic cellular systems

**Figure 4:**
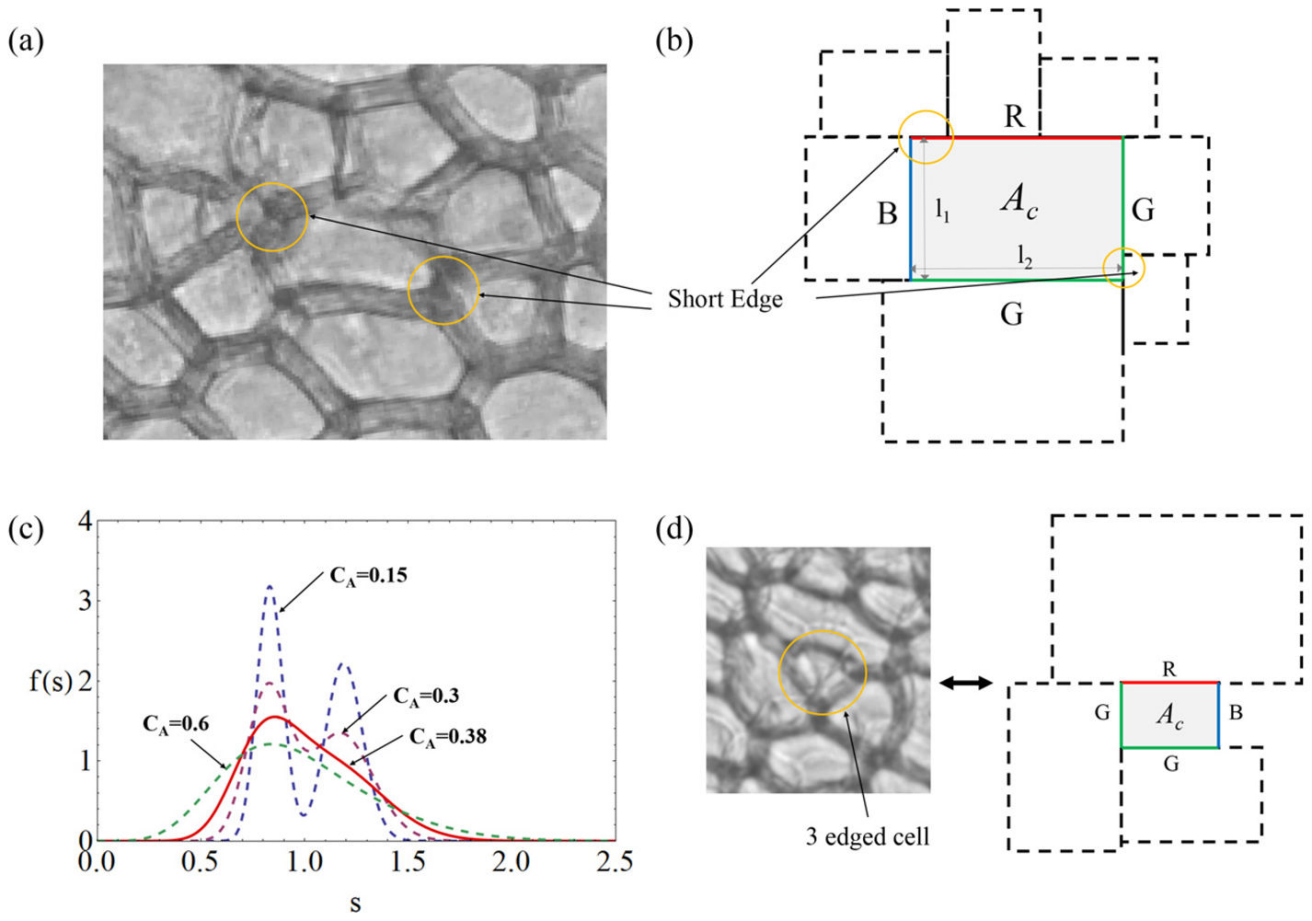
(a) Illustration of the occurrence of short-edge neighbors as a consequence of elongated (anisotropic) cell shape. (b) Schematic representation of the rectangular-cell model, with the three different types of edges (R, G and B) indicated by colors. The cells are polydisperse, but have the same aspect ratio. Short edges are highlighted in (a) and (b). (c) Probability distributions *f* (*s*) of edge lengths for area (gamma) distributions of different *c_A_*. (d) Example of a rare cell with *n* = 3 neighbors, corresponding to a modeling situation with one short B edge with *n_i_* = 0.

The key idea of the present modeling is that anisotropic (elongated) domains, for a given polydispersity of areas, allow for a greater variety of neighbor configurations, as a neighboring cell of given area can take up varying portions of the perimeter of the central cell, depending on relative orientation. In particular, short edges shared by neighboring cells are more common in this situation, specifically for cells that touch in regions of strong curvature (figure 4(a)). A description of neighbor relations depending on the full relative positioning and orientation of the domains quickly becomes unnecessarily complicated, even if simple ellipses are chosen as templates. Instead, we propose a model that captures the gist of the short-edge neighbor occurrences and preserves the aspect ratio *α* as the main governing parameter.

In this approach, we replace the domains with *rectangles* of uniform *α*, with edge lengths *ℓ*_1_ and *ℓ*_2_, where $\ell_{1}=\alpha \ell_{2}=\sqrt{\alpha A}$ (figure 4(b)). Neighboring domains are assumed to have sides parallel to the central rectangle, and the short edges are now a consequence of neighboring domains extending beyond the end point of a central edge (figure 4(b)). Within this framework, and with a given area distribution *P*(*A*) (figure 3(a)), we now have to evaluate the probability of a central cell, with the area *A*_c_, to have *n* neighbors.

The number of neighbors *n* of the central cell is merely the sum of the number of neighbors *n_i_* of the four individual edges. Hence, the conditional probability *P*(*n*|*A*_c_) can be calculated from that of an individual edge, *P*(*n_i_*|*l*_c_). Denoting general edge lengths by *s*, the probability distribution *f* (*s*) can be derived from *P*(*A*) by assuming that the orientation of the neighboring domain is equally likely; i.e. for a given neighboring cell, there is 50% chance of *s* = *l*_1_ for the neighboring edge and 50% chance of *s* = *l*_2_. It then follows that $f(s)=\frac{s}{\alpha }P(\frac{s^{2}}{\alpha })+s\alpha P(s^{2}\alpha )$ (figure 4(c)). In general, *f* (*s*) is of bimodal shape where the peak values occur near $s=\sqrt{\alpha }$ and $s=1/\sqrt{\alpha }$; this is qualitatively different from the unimodal angular distribution *f* (*ϕ*) of the isotropic disc model.

### 4.1. Edge neighbor configurations

As Euler’s theorem requires *n̄* = 6, the average edge of any rectangle must have 1.5 neighbors. However, even disregarding differences in edge length, not all edges of the central cell are the same with respect to neighbor patterns. Figure 4(b) shows that there are three different types of edges in a rectangular tiling, depending on whether the lengths of neighboring rectangles precisely add up to that of the edge in question (denoted as a ‘blue edge’ B in the figure), whether only one end of the central edge is flush with the rectangular neighbor, while the last neighbor at the other end overshoots the central edge (‘green edge’, G), or whether an overshoot occurs at both ends of the central edge (‘red edge’, R). In a tiling of rectangles, these types of edges will all be encountered, and their relative frequency will depend on how the tiling is constructed (e.g. by successive agglomeration or growth of separated nuclei). We do not know the details of how the pattern of elongated cells in *Cucumis* is formed, nor does it correspond directly to a rectangular tiling. We have tried different relative statistical weights of the three types of edges, but found that the final results reported below change little (neighbor probabilities *P_n_* change by less than 1%). It is easy to see that, in the limit of monodisperse rectangles (*c_A_* → 0), the expected average number of neighbors is *n_i_* = 2 for R edges, *n_i_* = 1.5 for G edges and *n_i_* = 1 for B edges.

The following model was adopted as the most realistic within the framework of rectangular tilings: the occurrence of neighbors ‘flush’ with a corner of the central rectangle is not generic if there is freedom of neighbor placement. Therefore, we assume that one central edge must be an R edge, with overshoots at both ends, granting the most freedom of configuration. One of the other three edges is then chosen with equal probability; if it is the opposite edge to the first, it is also designated an R edge—the two others are then necessarily B edges, and the edge pattern is RBRB. If the second edge is one of the adjacent edges to the first, it is designated a G edge (most freedom of neighbor placement, as R is not possible). The other two edges must then be one G and one B edge; we have to distinguish, however, between the configuration RGBG, where the two G edges are opposite each other (and thus of the same length), and RGGB, where they are of unequal length. Overall, the three patterns RBRB, RGBG and RGGB then occur with equal probability of 1/3.

### 4.2. Edge neighbor probabilities

We now calculate the probability of having *n_i_* neighbors for each edge type separately. For a G edge of length *l*_c_, tiling can be started at one vertex so the conditional probability *P*_G_(*n_i_*|*l*_c_) is the probability that the sum of *n* − 1 neighboring edges is less than *l*_c_ but longer than *l*_c_ for *n* neighboring edges, in a full analogy to the angular distribution of disc neighbors in [7]. Therefore using the notations $R_{n}(s)=P(\sum_{i=1}^{n}s_{i}=s)=\int_{0}^{s}f(\bar{s})R_{n-1}(s-\bar{s})\;d\bar{s}$ and $F(l_{c}-s)=\int_{l_{c}-s}^{\infty }f(\bar{s})\;d\bar{s}$, we obtain

(2) $$P_{G}(1|l_{c})=F(l_{c}),$$

(3) $$P_{G}(n_{i}|l_{c})=\int_{0}^{l_{c}}R_{n_{i}-1}(s)F(l_{c}-s)\;ds.$$

Calculating the conditional probability for R edges, *P*_R_(*n_i_*|*l*_c_), involves integration over an additional degree of freedom. After the length of the first neighboring edge, *s*_1_, is chosen, we also need to choose its location relative to the central edge, which we call *s̃* here (measured from the starting point of the central edge). We assume that *s̃* is uniformly distributed in the allowed range 0 ≤ *s̃* ≤ *s*_1_. When *n_i_* = 1, the length of the first neighboring edge should be larger than *l*_c_ and *l*_c_ ≤ *s̃* ≤ *s*_1_, so *P*(1|*l*_c_) is written as follows:

(4) $$P_{R}(1|l_{c})=\int_{l_{c}}^{\infty }f(s_{1})\frac{s_{1}-l_{c}}{s_{1}}ds_{1}.$$

When *n_i_* > 1, the first neighboring edge can be any length, but the allowed range of *s̃* is now 0 ≤ *s̃* ≤ *s*_1_ for *s*_1_ < *l*_c_, and 0 ≤ *s̃* ≤ *l*_c_ for *s*_1_ > *l*_c_. After placing the first cell, the conditional probability of *n_i_* − 1 neighbors for the remaining length *l*_c_ − *s̃* is the same as for a G edge, *P*_G_(*n_i_* − 1|*l*_c_ − *s̃*). Hence, *P*_R_(*n_i_*|*l*_c_) can be written in the following double integral form:

(5) $$P_{R}(n_{i}|l_{c})=\int_{0}^{l_{c}}ds_{1}\frac{f(s_{1})}{s_{1}}\int_{0}^{s_{1}}d\tilde{s}\;P_{G}(n_{i}-1|l_{c}-\tilde{s})+\int_{l_{c}}^{\infty }ds_{1}\frac{f(s_{1})}{s_{1}}\int_{0}^{l_{c}}d\tilde{s}P_{G}(n_{i}-1|l_{c}-\tilde{s}).$$

The conditional probability of a B edge cannot rely on the neighboring edge lengths exactly adding up to the central edge length (this would be a probability of zero). In reality, too-large neighboring cells would be ‘squeezed’ into a gap of length *l*_c_ and thus made to conform to the given edge length. At the same time, this squeezing cannot be assumed to work for arbitrarily large neighboring edges: If the neighboring edge is larger than *βl*_c_, where *β* > 1 is a constant, we take squeezing to be impossible, and do not count a further neighbor (the gap left would instead be interpreted as closed by the remaining neighbors, see figure 4(d); note that this mechanism provides for a—very small—probability of a cell having less than four neighbors, which does occur in our experiments for less than 0.1% of all cells). In summary, we assign probabilities as follows:

(6) $$P_{B}(0|l_{c})=F(\beta l_{c}),$$

(7) $$P_{B}(1|l_{c})=P_{G}(2|l_{c})+P_{G}(1|l_{c})-P_{B}(0|l_{c}),$$

(8) $$P_{B}(n_{i}|l_{c})=P_{G}(n_{i}-1|l_{c}).$$

To wit, this means that a B edge of length *l*_c_ is counted as having *n_i_* = 0 if the (first) neighboring edge is longer than *βl*_c_. It has *n_i_* = 1 if the situation is equivalent to fitting two neighbors at a G edge (*P*_G_(2|*l*_c_)) *or* if a single neighbor fits by squeezing (*P*_G_(1|*l*_c_) − *P*_B_(0|*l*_c_)). For *n_i_* ≥ 2, the B edge is exactly like a G edge except the last neighbor is not counted. It remains to determine the coefficient *β*. Rather than choosing it arbitrarily, we can make use of Euler’s theorem: when evaluating the average number of neighbors from adding contributions to all edges, we obtain a value that (weakly) depends on *β*—but we know that *n̄* = 6 must hold. Thus, the relation specifies a certain *β*(*α*, *c_A_*), of which we report the value below.

**Figure 5:**
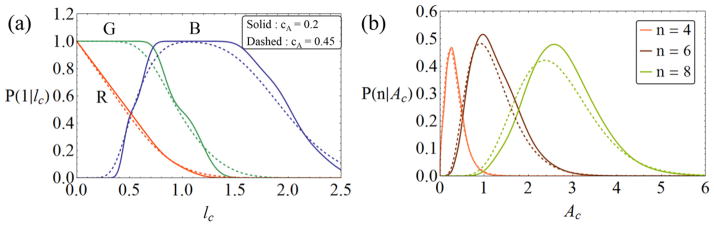
(a) Conditional probability of a central edge having one neighbor (*n_i_* = 1), given *l*_c_, for the R, G and B edges. The results differ little for significantly different *c_A_*. (b) Conditional probability of a cell of area *A*_c_ having *n* = 4, 6 or 8 neighbors. In both figures, solid lines: *c_A_* = 0.2, dashed lines: *c_A_* = 0.45.

Figure 5(a) shows that the conditional probabilities of each type of edge are indeed substantially different, especially for *n_i_* = 1, while the dependence on *c_A_* is not very pronounced.

### 4.3. Conditional and unconditional cell neighbor probabilities

To obtain *P*(*n*|*A*_c_), we now take the sum of the products of the conditional probabilities of the corresponding edges for all possible combinations such that $\sum_{i=1}^{4}n_{i}=n$, where *n_i_* is the number of neighbors of the *i*th edge, and also taking into account the equal probabilities of configurations RBRB, RGGB and RGBG (see section 4.1). The explicit formulas for this tedious but finite exercise in combinatorics are given in the appendix. We present the sample results in (b) for *n* = 4, 6 and 8. *P*(*n*|*A*_c_) has a bell shape curve that is well approximated by a normal distribution. The width of the curve becomes larger as n increases (figure 5(b)), because there is a larger number of combinations that obtain $\sum_{i=1}^{4}n_{i}=n$ for a larger value of *n*.

Finally, we compute the unconditional probabilities of having *n* neighbors as *P_n_* = ∫ *P*(*n*|*A*_c_) *P*(*A*_c_) d*A*_c_, as well as the average *n̄* = Σ*_n_ n P_n_*. From the requirement *n̄* = 6, we can find a value of *β* for each *c_A_* and *α*. Although there is variation of *β* values as *c_A_* and *α* change, the range of *β* is between 1.3 and 1.9 even for extreme cases outside the range of our present experimental data (we explored *α* as small as 0.44, and *c_A_* as large as 0.6). For the *Cucumis* cell samples relevant here, *β* only varies between 1.75 and 1.88.

**Figure 6:**
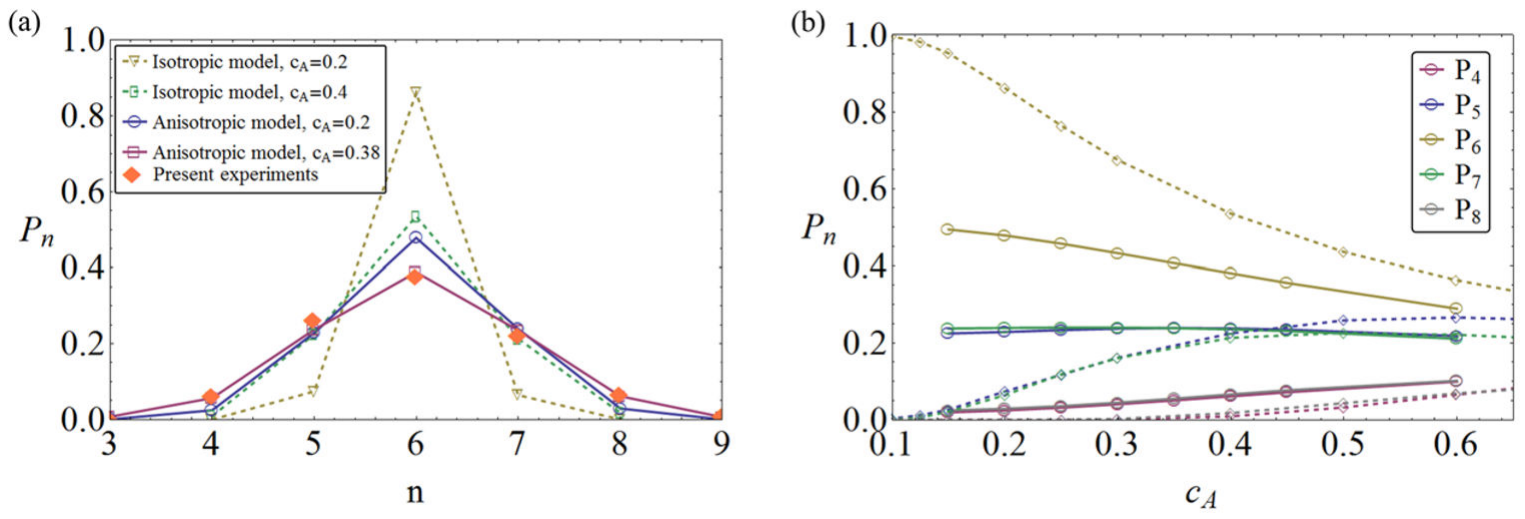
(a) Comparison of neighbor probabilities *P_n_* from experiment (solid symbols), the present anisotropic theory (open symbols, solid line), and the isotropic disc theory (open symbols, dashed line). Agreement with the anisotropic model at the experimentally observed *c_A_* = 0.38 is very good. (b) Dependence of *P_n_* on *c_A_* for the anisotropic and isotropic models. The former shows none of the crystallization features of the latter.

The neighbor distributions *P_n_* themselves are strikingly dissimilar from the isotropic (disc) case. Figure 6(a) shows that, for the same polydispersity (value of *c_A_*), *P_n_* is much wider in the anisotropic case. Plotting the *P_n_*(*c_A_*) dependence in full (figure 6(b)), we also see that the anisotropic model fails to show the typical crystallization-threshold effect of the isotropic case [7, 22]. While monodisperse discs will be strictly hexagonally ordered (*P*_6_ → 1), this is not true for the anisotropic objects, where the *orientation* of the rectangles always provides a variety of possible neighbor configurations. In this sense, anisotropic shape has to be accounted for as a *third* source of disorder: apart from size disorder and positional disorder [9], there is also *orientational disorder*, and its effect is clearly seen in the present study.

## 5. Results and discussion

We are now in a position to compute the main variables of size–topology correlation. Taking the aspect ratio *α* and the width *c_A_* of the gamma distribution (1) as inputs, the conditional probabilities *P*(*n*|*A*) and unconditional *P_n_* are computed as outlined above, with the integrations performed numerically. The neighbor distribution coefficient of variation *c_n_* then follows directly from

(9) $${\left(c_{n}\bar{n}\right)}^{2}=\sum_{n}P_{n}{\left(n-\bar{n}\right)}^{2}.$$

The average area of cells having *n* neighbors *Ā_n_*, on the other hand, is computed using Bayes’ theorem to obtain *P*(*A*_c_|*n*) from *P*(*n*|*A*_c_). Explicitly, we get

(10) $${\bar{A}}_{n}=\frac{1}{P_{n}}\int_{0}^{\infty }A_{c}P(n|A_{c})P(A_{c})\;dA_{c}.$$

**Figure 7:**
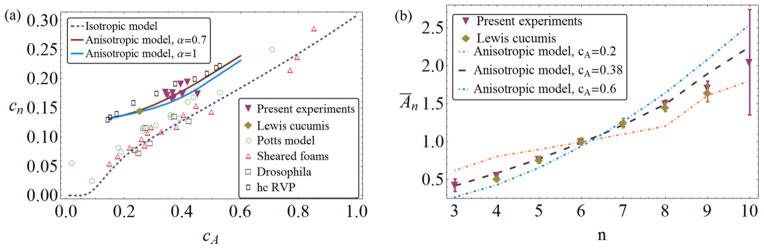
(a) Graph of *c_n_* – *c_A_* size–topology correlation with data and isotropic theory as in figure 2(c). The results from the present anisotropic theory (solid line) are consistent with both the current and Lewis’ data on *Cucumis*. (b) Lewis’ law *Ā_n_*(*n*) from the anisotropic theory (dashed lines) agrees with the experimental results when the experimental polydispersity *c_A_* = 0.38 is used in the theory. The curve changes significantly as *c_A_* is increased or decreased. Error bars are 95% confidence intervals.

The resulting *c_n_* value of our model significantly deviates from that of the disc model for a given *c_A_* and the simulation results show a good match with our experimental data of cucumbers (figure 7(a)). Incorporating significant anisotropy into the model indeed leads to larger *c_n_* at given *c_A_*, and quantitatively explains the observed deviations from the isotropic (disc) theory. Size disorder and orientational disorder cooperate to push *c_n_* to larger values. Note that the rectangular model does *not* revert to the disc results as the aspect ratio *α* approaches one (figure 7(a)), as the nature and importance of short-edge neighbors like those depicted in figures 4(a) and (b) does not diminish in this limit. Larger *α* does decrease *c_n_*, but not to the extent of disc isotropy. The model’s relative insensitivity to changes of *α* confirms that using one universal value *ᾱ* is a reasonable approximation.

As was observed above when discussing the *P_n_* distributions, crystallization does not happen in the anisotropic tilings, so that *c_n_* does not approach zero as *c_A_* → 0. The orientational disorder is sufficient to maintain a finite *c_n_* in all cases.

Finally, let us discuss the results for Lewis’ law, *Ā_n_*(*n*). Figure 7(b) shows that the values obtained from the model agree very well with both sets of experiments for the experimentally established range of 4 ≤ *n* ≤ 9, with no free parameters (note *β* was fixed by Euler’s theorem). Comparisons outside that range must await more extensive experimental data: among the over 4200 cells of our study, only four had *n* = 3 and only three had *n* = 10. The shape of Lewis’ law, however, is less universal than the analogous relation in the isotropic model: For the disc model, it was shown that *Ā_n_* has almost no *c_A_* dependence and thus provides a universal size law for both small and large polydispersities. For the rectangle model, we see significant variation of the shape of *Ā_n_* with *c_A_* (figure 7(b)). While the relation stays approximately linear, its slope decreases with decreasing *c_A_*.

How closely does our result approach the assumption of a linear Lewis law? If a linear law is assumed *a priori*, experimental or theoretical data conform to a one-parameter fit, as the constraint Σ*_n_ P_n_ Ā_n_* = 1 must be observed. The resulting relation can e.g. be written as [15]

(11) $${\bar{A}}_{n}=\frac{n-n_{0}}{6-n_{0}}$$

with the single parameter *n*_0_, translating into a slope *k* = 1/(6 − *n*_0_). The simplest argument then postulates that *A*_2_ = 0 (arguing that, as two-edged cells are not observed, they should have zero area), resulting in *n*_0_ = 2, which gives the linear law a slope of *k* = 0.25. This comes quite close to the best fit of our experimental data to (11), which results in *k*_exp_ = 0.235 (*R*^2^ ≈ 0.987). The model with *c_A_* = 0.38 obtains *k*_theo_ = 0.265. Not only does the model agree well with experimental data (dashed line in figure 7(b)) but it is also close to a straight line (*R*^2^ ≈ 0.962). Thus, our result mimics closely a law obtained when linearity is assumed, but does not itself assume linearity (and in fact, produces significant nonlinearities for larger *c_A_* values).

We have checked for further modeling dependences by replacing the Gamma distribution of (1) by a normal distribution of the same mean and variance. Such a replacement changed *Ā_n_* very little for the disc model [7]; in the present anisotropic model, deviations show up, but only for high *n* ≥ 10. If better statistics from experiment could be obtained, it is conceivable that such deviations could be used to probe the large-area tail of the *P*(*A*) distribution. For our present sample sizes, however, we do not have sufficient statistics to say whether the decay of *P*(*A*) at *A* ≫ 1 is better described by a Gamma or normal distribution. For the core range of the Lewis law (*n* between 4 and 9), in any event, our theory reproduces the experimental data robustly and quantitatively. The linearity of this part of *Ā_n_* may simply be a result of the adjustments made by the presence of orientational disorder in the system. Compared to the isotropic case, a large number *n* > 6 of neighbors can be accommodated in a larger number of situations, in particular those with smaller *A*, and thus higher probability. This tends to decrease *Ā_n_* for *n* > 6 over the disc case. Conversely, a small number of neighbors can occur now for larger central cells (if neighbor orientation is favorable), and this tends to increase *Ā_n_* for *n* < 6. This answers the questions concerning naive neighbor counting raised in the introduction: orientational disorder ensures that the assumption of ‘average’ neighbors is not valid, and the dependence of central cell area on *n* is less than quadratic.

In the isotropic disc model, it was possible to derive simple analytical expressions for the main results of the computation, largely because a formalism that replaced all distributions with normal distributions of equal mean and width proved accurate. For the present anisotropic model, such an approach may be less appropriate for reasons explained above. It would also result in considerably more complicated formulae, as there is no compact way of writing the combinatorial expressions that lead from the *n_i_* probabilities to the *n* probabilities. However, it is worthwhile mentioning here that accurate analytical representations using normal distributions can be obtained for all *P_ξ_* (*n_i_*|*l*_c_) (*ξ* = R, G and B), by approximating *R_n_*(*s*) by a normal distribution and *f* (*s*) by the sum of two normal distributions, preserving first and second moments in all cases. This can speed up computations for large-scale parameter scans.

## 6. Conclusions

We have shown that the fundamental correlations of domain size and neighbor number statistics are significantly different for anisotropic objects compared to isotropic domains in 2D tilings. Experimentally, this is confirmed by revisiting Lewis’ experiments with cucumber epidermis tissue and recognizing that the cells have a typical degree of anisotropy. Developing a new model for such anisotropic elements, we approximate the individual cell domains by rectangles and take into account the varying degrees of freedom in the placement of neighboring cells covering edges of a central cell. Like the disc model of isotropic domains, this new approach has only one parameter, which is fixed by the application of Euler’s theorem, ensuring that this local model of a single central cell and its neighbors has statistics compatible with plane-filling tilings.

The results show that anisotropy of the type and magnitude observed in experiment is a sufficient ingredient to explain both the differences in the *c_n_* – *c_A_* correlation curve and in the Lewis law plot, including the approximately linear shape of *Ā_n_* in the anisotropic case, in striking contrast to the nonlinear Lewis law expected and confirmed for systems of isotropic elements. It can be conjectured that there is a range of ‘Lewis laws’ interpolating between linear and nonlinear depending on the shape of tiling elements, and comprising the many examples found in experiment and simulation [15, 16, 20, 24, 30].

As the anisotropy parameter (aspect ratio) of the individual cells shows a pronounced peak at a certain value in our *Cucumis* system, we have simplified the model assuming all cells have the same aspect ratio. The approach could be refined by introducing a continuous aspect ratio distribution according to experimental data, adding one more integration variable for the averaging. Even in its present form, however, the model demonstrates the importance of orientational disorder in addition to size and positional disorder, and once again emphasizes that many statistical properties of a plane-filling ensemble can be understood from a local template of neighboring cells. The combination of anisotropy and size disorder is sufficient to explain the present (cucumber) data, without the need to explicitly evaluate positional disorder.

In our approach, we have not attempted to explain the physical or biological causes of the cell anisotropy. In [7], it was argued that the disc model is a good approximation for any 2D cellular system with prominent interfacial energy, where the individual domains are compelled by energy minimization to assume compact (isotropic) shapes of aspect ratios near one and without favored directions. Foams are a prime example of this type of system [31–37]. In the case of cucumber tissue, it stands to reason that a different energy contribution favors the anisotropic shape and partially compensates for interfacial elasticity that, by itself, would lead to roughly circular cells. Forces of the cytoskeletal bulk [38], cell wall stiffness [39], placement of cell organelles [40], differential adhesion forces [41–43] or overall morphological dynamics of the tissue [44] could be factors causing anisotropy in this way. Concerning the latter speculation, we did test for correlations of anisotropy in our cell samples (i.e. are the directions of the long axes of neighboring cells correlated?), but did not find conclusive signatures of large-scale organization. The exploration of causes of cell anisotropy is left to future investigation, which can now complement the link of cell shape and neighbor statistics established in the present work.

## Appendix. Calculation of the conditional neighbor probabilities

In section 4.2, we give closed expressions for the single-edge probabilities *P_ξ_*(*n*|*l*_c_), where *ξ* = R, G and B. From these, we first compute *P_ξη_*(*n*|*l*_c_), which is the conditional probability that two *parallel* edges of the central rectangle have *n* neighbors in total. Of these, there are six different types, i.e. *ξη* = RR, GG, BB, RG, RB and BG. Elementary combinatorics gives

(A.1) $$P_{\xi \eta }(n|l_{c})=\sum_{i+j=n}P_{\xi }(i|l_{c})P_{\eta }(j|l_{c}).$$

In the next step, we construct the probabilities that a particular rectangle has *n* neighbors, *P_ξεηψ_*(*n*|*A*_c_), from these *P_ξη_*(*n*|*l*_c_) by taking into account all cases for which the sum of the neighbors of the two long parallel edges and that of the two short parallel edges is equal to *n*. As explained in the main text, only three different ways of tiling the central cell exist, namely *ξεηψ* = RBRB, RGGB and RGBG. For any central rectangle, the short and long edge lengths are $l_{1}=\sqrt{A_{c}\alpha }$ and $l_{2}=\sqrt{A_{c}/\alpha }$, respectively, so the explicit formula can be written as follows:

(A.2) $$P_{\xi \varepsilon \eta \psi }(n|A_{c})=\frac{1}{2}\left[\sum_{i+j=n}P_{\xi \eta }\;\left(i|\sqrt{\frac{A_{c}}{\alpha }}\right)\;P_{\varepsilon \psi }(j|\sqrt{A_{c}\alpha })+\sum_{k+l=n}P_{\xi \eta }(k|\sqrt{A_{c}\alpha })P_{\varepsilon \psi }\;\left(l|\sqrt{\frac{A_{c}}{\alpha }}\right)\right].$$

Finally, *P*(*n*|*A*_c_) can be calculated from *P_ξεηψ_*(*n*|*A*_c_) with the assumption that each of the three types of tiling occurs with equal probability (see the discussion in the text),

(A.3) $$P(n|A_{c})=\frac{1}{3}P_{\text{RBRB}}(n|A_{c})+\frac{1}{3}P_{\text{RGGB}}(n|A_{c})+\frac{1}{3}P_{\text{RGBG}}(n|A_{c}).$$

With this conditional probability, the unconditional probabilities *P_n_* are obtained by convolution with *P*(*A*), as described in the main text.
